# Is lumbar spondylolisthesis a risk factor of cage subsidence after oblique lumbar interbody fusion combined with anterolateral screw fixation?

**DOI:** 10.1186/s13018-025-05624-3

**Published:** 2025-03-04

**Authors:** Xingrui Peng, Xiandi Wang, Tianhang Xie, Xiao Hu, Jiancheng Zeng

**Affiliations:** Department of Orthopedic Surgery and Orthopedic Research Institute, West China Hospital, Sichuan University, No. 37 GuoXue Rd, Chengdu, 610041 Sichuan China

## Introduction

Degenerative lumbar spondylolisthesis (DLS) and degenerative lumbar spinal stenosis (DLSS) are two of the most common degenerative spinal diseases among the middle and elderly aged people, which cause low-back pain, leg pain and functional disorder [1, 2]. For treating degenerative lumbar disease, multiple surgical techniques were developed. As was first reported by Silverstre et al. [3] in 2012, oblique lumbar interbody fusion (OLIF) technique was one of them. Compared to traditional posterior surgical approaches, OLIF preserves all the back structures, such as bone, ligaments, and paravertebral muscles, thereby maintaining the maximum sagittal balance of the lumbar spine [4]. Because of the advantage of preserving all the posterior structures and minimal invasion, OLIF was used widely for treating degenerative lumbar disease and developed rapidly in the past decade. And anterolateral screw fixation (AF) had been demonstrated to be effective in enhancing spinal stability [5]. However, OLIF combined with AF is not widely accepted yet.

CS is the one of the most common complications of OLIF postoperatively with the occurrence rate of 10–46.7% [6, 7]. In our previous study, OLIF-AF was successfully applied for the treatment of DLSS but the CS rate was noticeable [8]. As reported, risk factors of CS were low bone mineral density (BMD), cage height, cage position, age and body mass index (BMI) et al. [9]. However, the impact of lumbar spondylolisthesis on CS has never been discussed. The symptoms of DLS were caused by dynamic stenosis, which were similar to DLSS. And because spine is unstable and the stenosis is dynamic, the biomechanical environment of DLS is more complicated than DLSS. Though in our previous studies OLIF-AF was used to ensure biomechanical stability of the spine and proved effective [10, 11], AF has totally different stress transmission mode compared to pedicle screw fixation and the impact of lumbar spondylolisthesis on CS after OLIF-AF has never been discussed. Thus we started this research, by comparing outcomes especially CS rate between OLIF-AF treating DLS and DLSS to evaluate whether lumbar spondylolisthesis a risk factor of CS after OLIF-AF.

## Methods

This study, approved by the ethics committees of West China Hospital of Sichuan University, was a retrospective matched-pair case-controlled investigation (approval number: 2023 − 441). It included a total of 184 patients (42 males and 142 females) who underwent L4-L5 OLIF-AF between April 2019 and June 2021. A total of 92 patients diagnosed DLSS who matched DLS group in sex, age, BMI and BMD were included as DLSS group. The matching effectiveness was evaluated by standard mean difference (SMD).

### Inclusion and exclusion criteria

Inclusion criteria: (1) patients with single-level L4-L5 DLSS with instability without DLS of L4 vertebra for DLSS group; (2) DLS of L4 vertebra of Meyerding grade I or II for DLS group; (3) conservative treatment for over 3 months without experiencing relief from low-back pain or leg pain; (4) DEXA T value>-2.5 SD; (5) minimum follow-up period of 24 months. Instability of the lumbar spine was assessed on lumbar flexion-extension X-ray, and was defined as slippage of more than 4 mm or angulation change more than 10° [12].

**Fig. 1:**
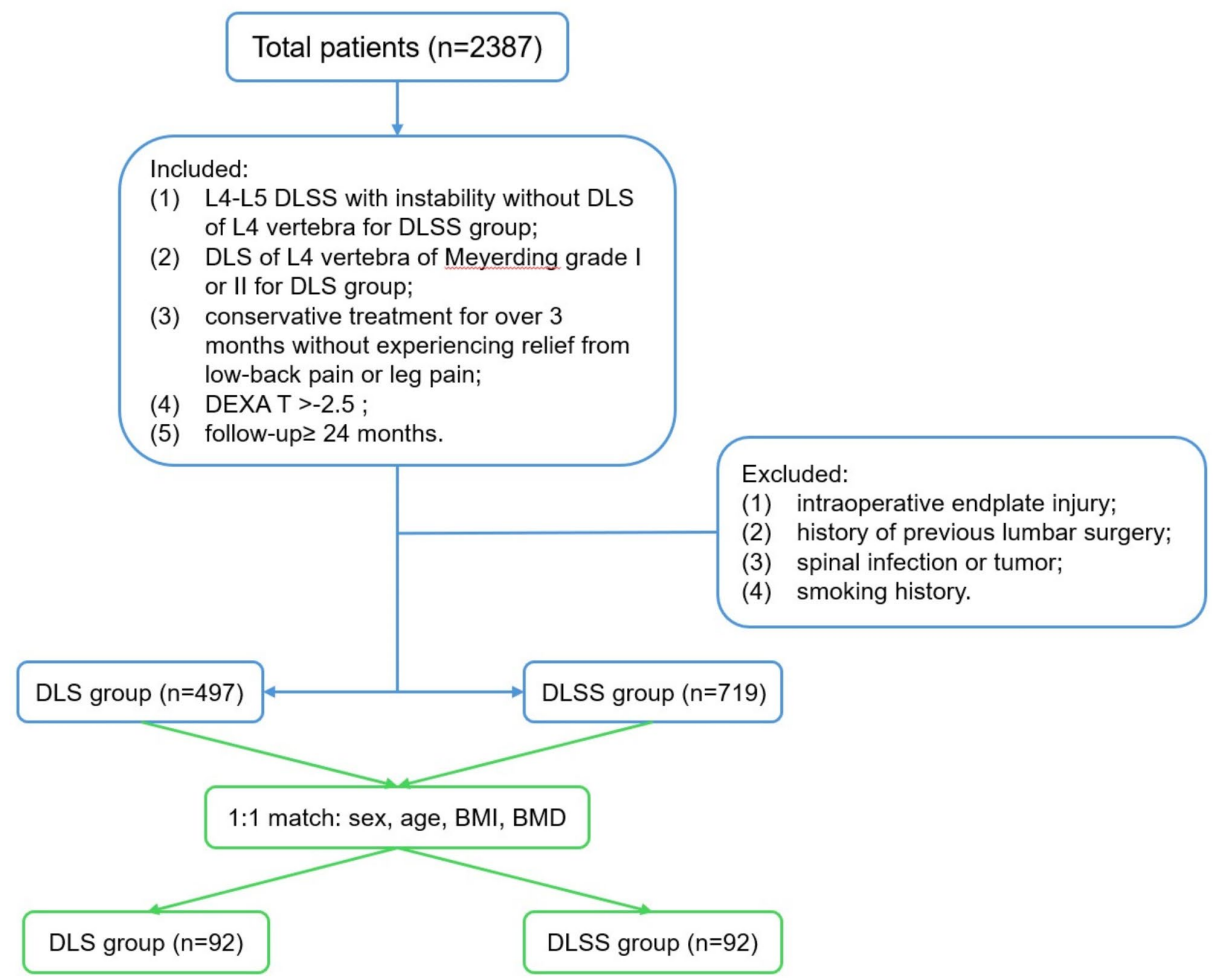
Flowchart of patient selection

Exclusion criteria: (1) intraoperative occurrence of endplate injury; (2) history of previous lumbar surgery; (3) spinal infection or tumor; (4) smoking history. (Fig. 1)

### Surgical methods

After the induction of general anesthesia and intubation, patients were positioned in the lateral decubitus posture, and C-arm fluoroscopy was used to locate the target intervertebral disc. A 5-cm incision was made in the lateral abdominal region, parallel to the iliac crest. Using a muscle-splitting technique, the external oblique, internal oblique, and transverse abdominal muscles were dissected in the direction of their fibers. Blunt dissection was performed to access the retroperitoneal space, and the peritoneal contents were mobilized anteriorly. The psoas muscle was identified and split at anterior 1/3. Then the anterior 1/3 of psoas muscle was tracked toward ventral side to expose the operation area and protect the ureter as well as major vessels. Once the L4-L5 intervertebral disc and the lateral side of the adjacent vertebral body were visualized, C-arm fluoroscopy was used to confirm proper placement before initiating interbody fusion. A tubular retractor system was then attached. Following discectomy, the vertebral endplates were prepared for cage insertion. A cage filled with synthetic bone containing bone morphogenetic protein-2 (BMP-2) was inserted into the exposed disc space. Vertebral screws were inserted and secured on the lateral side of the vertebral body and close to the vertebral endplate. A connecting rod was placed and locked. Finally, the abdominal muscle planes and skin were sequentially closed. All patients strictly wore lumbar brace for 3 months postoperatively.

### Radiological measurements

Preoperatively, as well as 1 day and 24 months postoperatively, lumbar X-ray and three-dimensional computed tomography (3D-CT) scans were collected. Disc height (DH) was defined as our previous research [13]. The change in DH (ΔDH) was calculated using the formula: ΔDH = (DH at 24 months postoperatively - DH at 1 day postoperatively). ΔDH greater than 2 mm after the operation was defined as CS. ΔDH ranging from 2 mm to 4 mm was classified as mild CS, while it exceeding 4 mm was considered severe CS. Prior to the operation, dual-energy X-ray absorptiometry (DEXA) was performed to assess bone mineral density (BMD), specifically the minimum T value of the hip was recorded. The fusion status was evaluated using 3D-CT scans, and the fusion grade criteria followed the guidelines set by Bridwell et al. [14]. Grade 1 and 2 were indicative of fusion. Two authors performed independent measurements of the remaining continuous variables, and the resulting values were averaged.

### Clinical evaluation

All study participants completed a minimum of 24 months of follow-up. Information such as sex, age, BMI, operation duration, intraoperative blood loss and hospital stay was documented. Preoperatively, as well as 3 and 24 months after the operation, the visual analog scale (VAS) scores for low-back pain (VAS-LBP) and leg pain (VAS-LP), as well as the Oswestry Disability Index (ODI), were recorded. Follow-up was conducted through clinic visits and telephone interviews. Surgical-related complications were carefully documented.

### Statistical analysis

Data analysis was performed using GraphPad Prism 10.1.2 software (GraphPad Software, CA, USA). Numerical continuous variables were presented as mean ± standard deviation. Age, BMI, BMD, operation duration, intraoperative blood loss and length of hospitalization were analyzed using t-test. Differences in CS rate and fusion status were compared using chi-square test. Changes in DH, VAS-LBP, VAS-LP, and ODI scores before and after the operation were evaluated using two-way ANOVA. *P*-value less than 0.05 was considered statistically significant.

## Results

### General data

A total of 184 patients who met the inclusion and exclusion criteria were included for the study. In the DLS group, there were 21 males and 71 females. Their age, BMI, and BMD were 62.7 ± 6.4 years, 22.6 ± 1.7 kg/m^2^, and − 1.8 ± 0.4 SD, respectively. Blood loss, operation time, and hospitalization time were 33.7 ± 7.7 ml, 96.1 ± 12.1 min, and 5.7 ± 1.4 days, respectively. In the DLSS group, there were 21 males and 71 females. Their age, BMI, and BMD were 63.0 ± 6.8 years, 22.8 ± 1.6 kg/m^2^, and − 1.8 ± 0.5 SD, respectively. Blood loss, operation time, and hospitalization time were 31.8 ± 7.0 ml, 95.3 ± 13.3 min, and 5.4 ± 1.3 days, respectively. Statistical analysis showed no significant difference between the two groups. And cage sizes of two groups were recorded. (Table 1)

**Table 1:** Comparison of general data between two groups

	DLS (*n* = 92)	DLSS (*n* = 92)	*p*	SMD
Sex (male: female)	21:71	21:71		
Age (year)	62.7 ± 6.4	63.0 ± 6.8	0.696	-0.052
BMI (kg/m^2^)	22.6 ± 1.7	22.8 ± 1.6	0.521	-0.086
BMD (T value SD)	-1.8 ± 0.4	-1.8 ± 0.5	0.561	-0.074
Operation duration (min)	96.1 ± 12.1	95.3 ± 13.3	0.661	
Blood loss (ml)	33.7 ± 7.7	31.8 ± 7.0	0.068	
Hospitalization (d)	5.7 ± 1.4	5.4 ± 1.3	0.129	
Cage size(mm)				
8*50 10*45 10*50 10*55 12*45 12*50 12*55 14*50 14*55	1 18 12 6 9 22 14 6 4	3 16 18 4 9 23 10 5 4		

BMD, bone mineral density; BMI, body mass index; SMD, standard mean difference

Data presented as mean ± standard deviation

### Radiologic evaluation

**Fig. 2:**
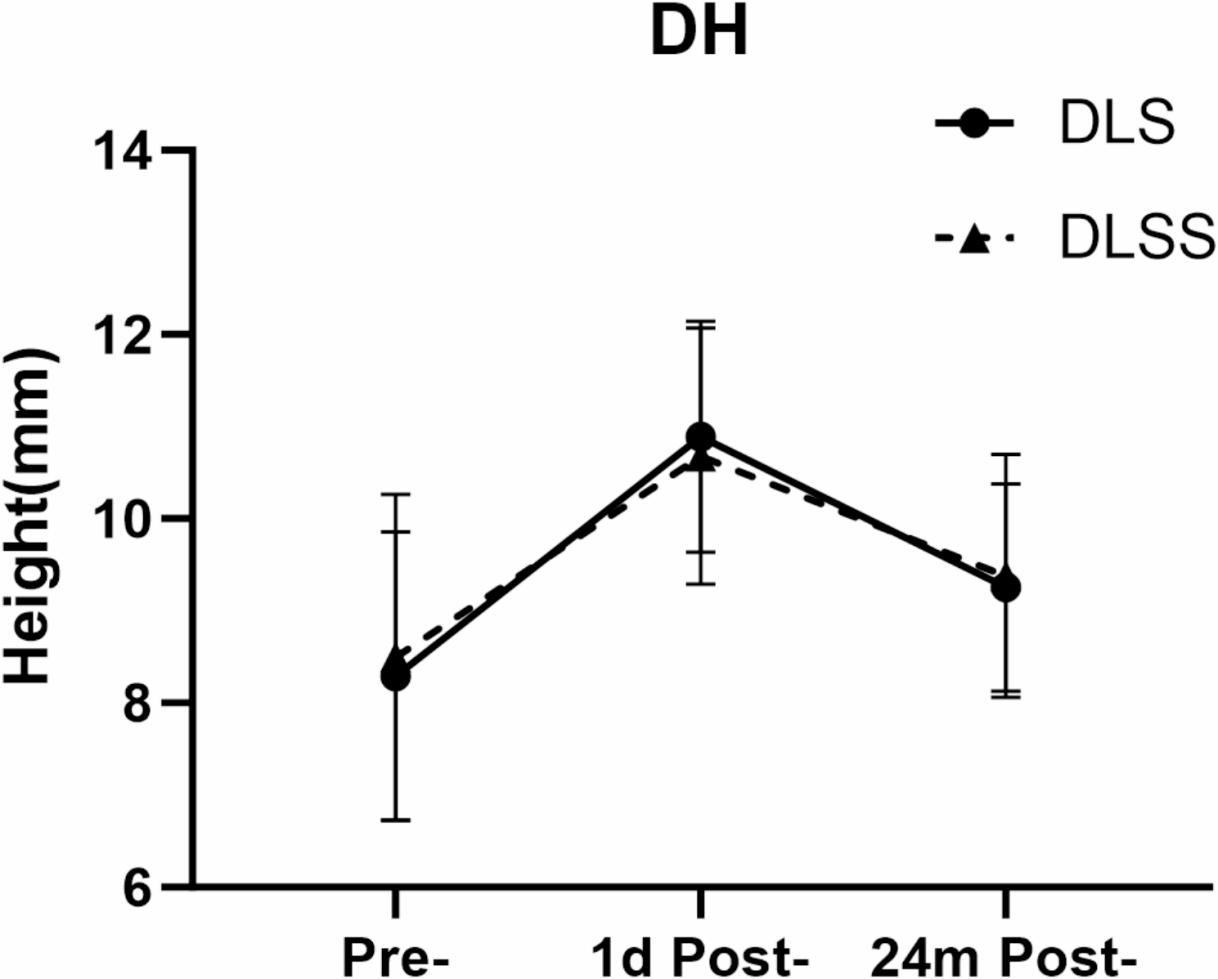
DH, disc height

In the DLS group, DH increased significantly from 8.29 ± 1.57 mm preoperatively to 10.89 ± 1.25 mm (*p* < 0.05) 1 day after the surgery. Subsequently, it decreased to 9.25 ± 1.12 mm (*p* < 0.05) at 24 months postoperatively. In the DLSS group, DH increased significantly from 8.50 ± 1.77 mm preoperatively to 10.68 ± 1.39 mm (*p* < 0.05) 1 day after the surgery. Then it decreased to 9.38 ± 1.32 mm (*p* < 0.05) at 24 months postoperatively. Additionally, average change in DH (ΔDH) in the DLS group was higher than the DLSS group (1.61 ± 0.93 mm vs. 1.30 ± 0.86 mm, *p* < 0.05). (Table 2; Fig. 2)

**Table 2:** Comparison of radiographic parameters between two groups

	DLS (*n* = 92)	DLSS (*n* = 92)	*p*
DH (mm)			
Pre-	8.29 ± 1.57	8.50 ± 1.77	0.790
1d Post-	10.89 ± 1.25§	10.68 ± 1.39§	0.632
24 m Post-	9.25 ± 1.12§†	9.38 ± 1.32§†	0.867
ΔDH	1.61 ± 0.93	1.30 ± 0.86	< 0.05*

DH, disk height; ΔDH, change of DH between 1 day postoperatively and 24 months postoperatively; Pre-, preoperative; post-, postoperative; n, number of patients

Data presented as mean ± standard deviation

* statistical significance between two groups

§ *p* < 0.05 compared to pre-

† *p* < 0.05 compared to 1 day post-

**Fig. 3:**
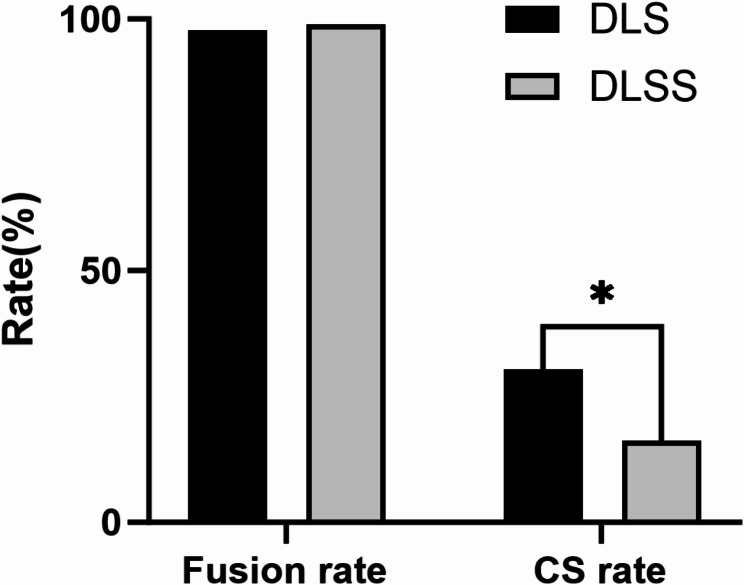
CS, cage subsidence. *p < 0.05 for the comparison between the two groups

Among the patients in the DLS group, 28 cases (30.43%) experienced CS, and all of them were mild. In the DLSS group, CS occurred in 15 cases (16.30%) and all were mild. No severe CS cases were observed in both groups. CS rate was statistically significant different between two groups (*p* < 0.05). However, no statistically significant difference of fusion rate between two groups was found (*p* > 0.99). (Table 3; Fig. 3)

**Table 3:** Comparison of CS rate and fusion rate between two groups

	DLS (*n* = 92)	DLSS (*n* = 92)	*p*
CS (n)			< 0.05*
None	64	77	
CS	28(30.43%)	15(16.30%)	
Fusion (n)			> 0.999
None	2	1	
Fusion	90(97.83%)	91(98.91%)	

CS, cage subsidence; n, number of patients

* statistical significance between two groups

### Clinical and functional evaluation

**Fig. 4:**
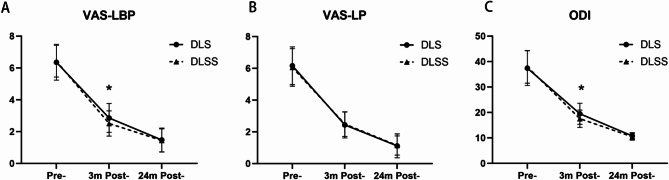
VAS-LBP, Visual Analog Scale score of the low-back pain; VAS-LP, Visual Analog Scale score of leg pain; ODI, Oswestry Disability Index at different time points for the two groups. *p < 0.05 for the comparison between the two groups

The DLS group showed significant improvement in VAS-LBP and VAS-LP scores after the surgery. The VAS-LBP decreased from 6.36 ± 1.13 to 2.86 ± 0.91 (*p* < 0.05) at 3 months postoperatively and further decreased to 1.48 ± 0.75 (*p* < 0.05) at 24 months postoperatively. Similarly, the VAS-LP decreased from 6.16 ± 1.19 to 2.44 ± 0.82 (*p* < 0.05) at 3 months postoperatively and further decreased to 1.12 ± 0.75 (*p* < 0.05) at 24 months postoperatively. In the DLSS group, significant improvements were also observed. The VAS-LBP decreased from 6.42 ± 1.00 to 2.51 ± 0.79 (*p* < 0.05) at 3 months postoperatively and further decreased to 1.45 ± 0.73 (*p* < 0.05) at 24 months postoperatively. The VAS-LP decreased from 6.05 ± 1.18 to 2.49 ± 0.78 (*p* < 0.05) at 3 months postoperatively and further decreased to 1.14 ± 0.60 (*p* < 0.05) at 24 months postoperatively. When comparing the two groups, VAS-LBP of the DLS group was significantly higher than the DLSS group at 3 months after the surgery (*p* < 0.05; *p* < 0.05). (Table 4; Fig. 4A and B)

**Table 4:** Comparison of VAS and ODI scores between two groups

	DLS (*n* = 92)	DLSS (*n* = 92)	*p*
VAS-LBP			
Pre-	6.36 ± 1.13	6.42 ± 1.00	0.967
3 m Post-	2.86 ± 0.91§	2.51 ± 0.79§	< 0.05*
24 m Post-	1.48 ± 0.75§†	1.45 ± 0.73§†	0.987
VAS-LP			
Pre-	6.16 ± 1.19	6.05 ± 1.18	0.899
3 m Post-	2.44 ± 0.82§	2.49 ± 0.78§	0.955
24 m Post-	1.12 ± 0.75§†	1.14 ± 0.60§†	0.995
ODI			
Pre-	37.41 ± 6.83	37.91 ± 6.40	0.831
3 m Post-	19.48 ± 4.12§	17.54 ± 3.38§	< 0.05*
24 m Post-	10.75 ± 1.32§†	10.44 ± 1.30§†	0.950

VAS-LBP, Visual Analog Scale of low-back pain; VAS-LP, Visual Analog Scale of leg pain; ODI, Oswestry Disability Index; n, number of patients

Data presented as mean ± standard deviation

* statistical significance between two groups

§ *p* < 0.05 compared to pre-

† *p* < 0.05 compared to 3 months post-

In the DLS group, the ODI score was 37.41 ± 6.83 before the surgery. Following the operation, there was a significant improvement in the ODI score, which decreased to 19.48 ± 4.12 (*p* < 0.05) at 3 months and further improved to 10.75 ± 1.32 (*p* < 0.05) at 24 months postoperatively. Similarly, in the DLSS group, the ODI score decreased from 37.91 ± 6.40 before the surgery to 17.54 ± 3.38 (*p* < 0.05) at 3 months and further improved to 10.44 ± 1.30 (*p* < 0.05) at 24 months postoperatively. Comparing the two groups, ODI of the DLSS group was significantly lower than DLS group (*p* < 0.05). (Table 4; Fig. 4C)

### Complications

**Fig. 5:**
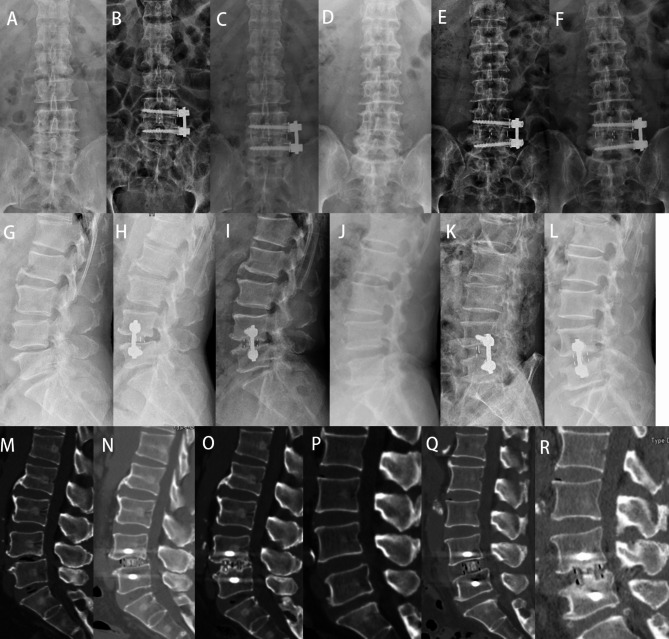
A patient of L4 DLS underwent OLIF-AF. (**A, B, C**) A-P X-ray preoperatively, 1 day and 24 months postoperatively; (**G, H, I**) lateral X-ray preoperatively, 1 day and 24 months postoperatively; (**M, N, O**) sagittal CT preoperatively, 1 day and 24 months postoperatively. CS occurred, no fixation loosening was observed and fusion was accomplished. A patient of L4-5 DLSS underwent OLIF-AF. (**D, E, F**) A-P X-ray preoperatively, 1 day and 24 months postoperatively; (**J, K, L**) lateral X-ray preoperatively, 1 day and 24 months postoperatively; (**P, Q, R**) sagittal CT preoperatively, 1 day and 24 months postoperatively. DH slightly deceased and CS did not occur, no fixation loosening was observed and fusion were all accomplished in both cases

There was no cerebrospinal fluid leakage, ureteral injury, major vascular injury, or nerve damage etc. occurred. After the surgery, 16 patients had pain in the left thigh and 2 patients had pain in the right thigh, and all fully recovered within 2 months. (Table 5; Fig. 5)

**Table 5:** Complications

	DLS (*n* = 92)	DLSS (*n* = 92)
Cerebrospinal fluid leakage	0	0
Ureteral injury	0	0
Major vascular injury	0	0
Nerve damage	0	0
Screw misplacement	0	0
Operation related infection	0	0
Left thigh pain	10	6
Right thigh pain	1	1
Cage subsidence	28	16

## Discussion

OLIF-AF has been shown to be effective in treating degenerative lumbar disease according to our previous studies [8, 10–11]. However, CS is one of the most common postoperative complications [7]. Risk factors of CS had already been widely discussed. As Park et al. [15] reported, osteoporosis, cage shape, cage position and endplate injury were the risk factors. Yao et al. [9] reported BMD and cage height as the significant risk factors. Kotheeranurak et al. [16] reported age and severe multifidus muscle fatty degeneration additionally. Except for the risk factors mentioned above, Singhatanadgige et al. [17] thought BMI and the use of demineralized bone matrix were correlated to CS. Several researches pointed out the morphology of the endplates affects CS occurrence after OLIF, and mismatch between endplates and grafted bone increases risk of screw loosening, which may also increase CS rate [18–20]. We think lumbar spondylolisthesis is a risk factor of CS followed OLIF-AF. And the results of this study supported our hypothesis.

It is crucial to prevent endplate damage. Throughout the procedure, the caudal endplates are easier to access but are also more vulnerable to damage. Additionally, a non-parallel trial fitting can result in direct endplate injury. Hence, we opted to retain a delicate cartilaginous endplate layer as a protective buffer before the trial fitting and subsequently removed it with care post-fitting. Furthermore, we observed that using a cage with a height greater than 14 mm in OLIF-AF significantly increases the CS rate. Therefore, the cages selected for the cases we used did not exceed a height of 14 mm.

DLS was characterized as the movement of one vertebra over the vertebra below, accompanied by degenerative alterations, without any accompanying disruption or flaw in the vertebral ring [21]. Further leading to instability, vertebra slippage, and subsequently segmental spondylolisthesis, osteophyte formation, facet joint arthritis, and ligament hypertrophy [22]. According to Wang et al., biomechanical study had shown a significant increase in shear stress between the slipped vertebrae in spondylolisthesis [23]. Since OLIF-AF cannot achieve complete reduction of the spondylolisthesis, we believe that this sustained increase in shear stress, deviating from the normal spinal alignment, may contribute to postoperative CS. Additionally, it has been reported that preoperative pelvic incidence (PI), segmental lordosis (SL), and lumbar lordosis (LL) angles are also associated with CS [24]. Improper correction of PI, SL, and LL may lead to excessive local shear stress postoperatively, potentially resulting in CS. Pedicle screw fixation mainly enhances posterior column of the spine [25]. AF mainly enhances anterior and middle column. The differences between the two internal fixation methods can lead to variations in the biomechanical environment, raising the question of whether lumbar spondylolisthesis is a risk factor for CS after OLIF-AF worth discussing.

According to previous researches, the CS rate after lumbar interbody fusion combined with pedicle screw fixation ranged from 10 to 46.7% [6, 7]. Our results showed the overall CS rate is about 23.4% (43/184), satisfactory pain and functional improvements were also achieved after OLIF-AF. Compared to pedicle screw internal fixation, OLIF-AF offers advantages as it does not require changing the patient’s position during surgery and can be completed through a single incision. Suggesting OLIF-AF has comparable effectiveness and even has certain advantages.

After OLIF, mild DH loss helps rematch of the cage and endplate, increasing the contact area and facilitating fusion. However, excessive DH loss can lead to CS and adversely affect indirect decompression of OLIF. A study by Marchi et al. [26] found that as the grade of CS increases, the severity of back pain increases. In this study, we found that the ΔDH and CS rates were higher in the DLS group. CS rate of DLS group was approximately 1.87 times of DLSS group. Additionally, the DLS group had higher VAS-LBP and ODI scores at 3 months postoperatively. However, no significant differences were observed between the two groups in the above-mentioned scores at 24 months postoperatively. Therefore, we believe that preventing CS is of significant importance for early postoperative pain relief and functional improvement.

The most commonly used method for preventing CS is bone cement augmentation, and our method was called Stress Endplate Augmentation (SEA) [8]. SEA was firstly reported by our team, which was applied to enhance load bearing area’s endplates by injecting bone cement into intraosseous trajectory followed by screw insertion. However, it is typically only applied to patients with osteoporosis (DEXA T <-2.5 SD). Whether should we widen the indication of applying SEA in DLS patients even their DEXA T >-2.5 SD still requires further research. Meanwhile, when considering mismatch of endplates and interbody fusion device, developing new type of cages and grafted material which can fulfill multiple morphological and biomechanical conditions may greatly reduce CS rate and accelerate fusion process. In addition to achieving thorough decompression and stable reconstruction through internal fixation, we should also properly reduce the spondylolisthesis to alleviate postoperative local stress concentration.

The core of our DH measuring method lies in the midpoint of the caudal vertebra endplate, making it suitable for Mayerding grade I and II of lumbar spondylolisthesis. If the spondylolisthesis reaches grade III or IV, this method will not be applicable, further exploration is needed to determine how to measure DH under such conditions.

However, there are several limitations that need to be considered in this study. Firstly, it is a retrospective study conducted at a single center with a relatively small sample size which may had selection bias. Secondly, although matching was performed, some unmeasured confounders may still exist, and the two groups were not fully matched in terms of cage sizes, while cage size has a significant impact on the occurrence of CS [14, 15, 27]. Thirdly, this study focused only on the L4-L5 segment. Fourthly, preoperative L4 spondylolisthesis of Mayerding grade I and II were not matched and discussed independently. Lastly, there is still lack of biomechanical evidence on the impact of lumbar spondylolisthesis on CS, as well as the effects of PI, LL, and SL on CS after OLIF-AF. Future researches should make efforts to address these limitations.

## Conclusions

This study demonstrates that OLIF-AF is safe and effective for treating both DLS and DLSS. However, the CS rate of DLS was significantly higher in the DLS group compared to the DLSS group, indicating that lumbar spondylolisthesis may be a risk factor of CS after OLIF-AF. And CS may lead to early postoperative pain and functional impairment.

## Data Availability

No datasets were generated or analysed during the current study.
